# Processes and determinants of integration of eGFR in physicians’ drug prescriptions: a qualitative study of semi-structured interviews

**DOI:** 10.1017/S1463423619000847

**Published:** 2019-10-23

**Authors:** Louis Lefèvre, Maud Jourdain, Jean-Pascal Fournier

**Affiliations:** Département de Médecine Générale, Faculté de Médecine, Université de Nantes, Nantes, France

**Keywords:** chronic, general practice, pharmacist, prescriptions, primary healthcare, qualitative research, renal insufficiency

## Abstract

**Aim:**

Our objective was to explore the processes and determinants leading physicians to integrate estimated glomerular filtration rate (eGFR) in their drug prescriptions

**Background:**

Access to patients’ eGFR would allow primary care pharmacists to optimise their role in the procedure of safe prescribing. Some rare physicians actively integrate eGFR in their prescriptions, in a sporadically and uncoordinated manner.

**Methods:**

Qualitative study using semi-directed interviews conducted among 12 French physicians who integrated eGFR in their drug prescriptions, (February 2016–April 2017). These voluntary participants were recruited through different means: Twitter®, forums, direct contact and snowball sampling. Data analysis was based on the grounded theory approach, underpinned by a comprehensive perspective of interactionist orientation.

**Findings:**

Residency and training, professional experience – including experiences of adverse drug reactions – and the membership in various communities of professionals were key drivers for the integration of eGFR in prescriptions. The theoretical aim was above all safe prescribing in order to reduce adverse drug reactions, with the control by a dispensing pharmacist and/or other healthcare professionals. Nevertheless, none of the physicians had received any feedback from any healthcare professionals. Despite their disappointment, the physicians remained convinced of the interest of integrating eGFR in their prescriptions and would continue to do so. Characteristics associated with integration of eGFR in drug prescriptions belong partly to Roger’s theory of innovations. If a widespread diffusion of this habit takes place, it will be necessary to evaluate its adoption by both physicians and pharmacists.

## Introduction

The prevalence of chronic kidney disease (CKD) is estimated of 4.9% in the UK, 13% in the US and is constantly on the rise worldwide (Lusignan *et al*., 2005; Coresh *et al*., 2007; Otero *et al*., 2010). Patients with CKD are at high risk of being exposed to drug prescription errors (20–67% of prescriptions) (Bhardwaja *et al*., 2011). Prescription errors even increase with the worsening of CKD (Breton *et al*., 2011). The increase in prescription errors would partly explain the increase in adverse drug reactions and admissions to hospital related to adverse drug reactions related to the latter that are observed in patients with CKD (Zhang *et al*., 2009).

Numerous studies have underlined the important role played by pharmacists in various interventions aiming at reducing inappropriate prescriptions and adverse drug reactions in patients with CKD (Hassan *et al*., 2009; Geerts *et al*., 2012; Via-Sosa *et al*., 2013).All these interventions rely on the prerequisite that the pharmacist has direct access to the patient’s estimated glomerular filtration rate (eGFR). French general practitioners are recognised as heavy prescribers, with around three out of four general practice encounters ending with a drug prescription (Cartier and Bourgueil, 2015). Prescription drugs can be dispensed by any independent primary care pharmacist (with the exception of methadone, buprenorphine and methylphenidate). Information technology systems for communication between primary care practices and pharmacies barely exist (Cartier and Bourgueil, 2015). Also, primary care pharmacists do not receive patients’ laboratory tests results. As such, French primary care pharmacists do not have direct access to the patient’s eGFR.

**Figure 1.**
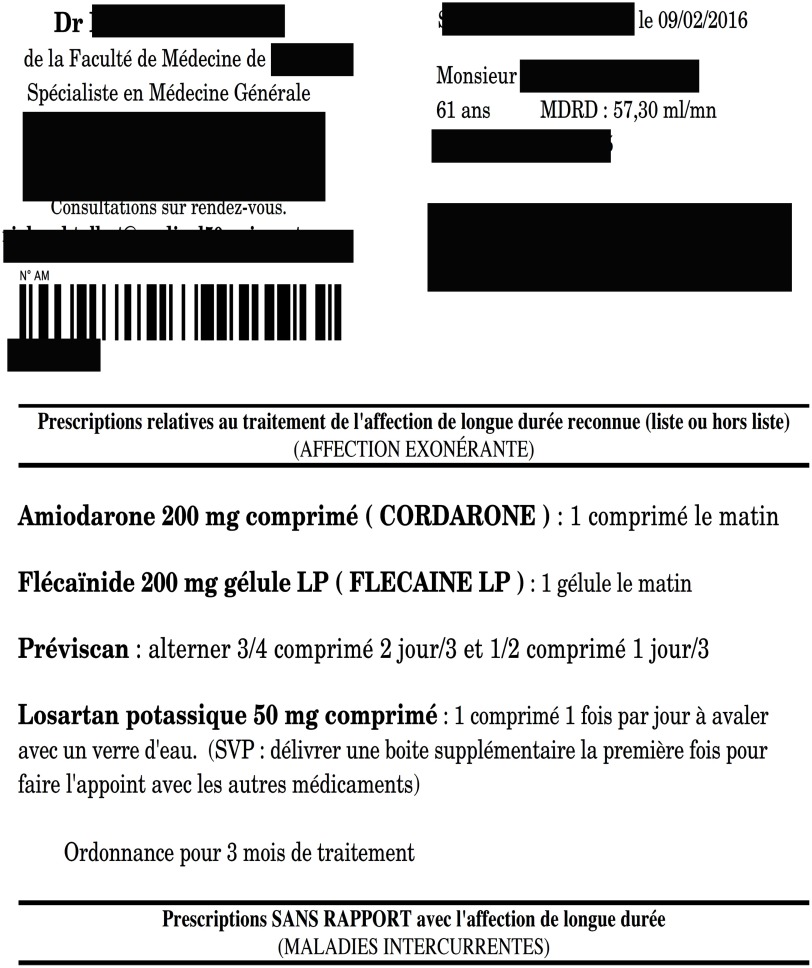
Example of a prescription drawn up by a physician interviewed

Since 2013 in the Netherlands, physicians (whatever their specialty) are legally obliged to provide the pharmacist with the drug indication, as well as ‘abnormal’ eGFR [Modification of Diet in Renal Disease (MDRD) equation <50 mL/min/1.73 m^2^] of the patients, for 23 specific drugs together with their drug prescriptions (The Royal Dutch Medical Association (KNMG), 2013). In France, there is currently no obligation to integrate any estimator of kidney function in a drug prescription (L’assurance maladie, 2017). Nevertheless, according to our experience in the field, some rare physicians (mostly primary care physicians) do integrate eGFR in drug prescriptions (Figure 1). We therefore attempted to understand this habit that some rare individuals have. According to our hypothesis, this particular phenomenon would broadly illustrate the issues of inter-professional cooperation that constitute a paradigm between primary care physicians and pharmacists.

We conducted an exploratory qualitative study using semi-structured interviews that aimed at exploring the process and determinants leading to the integration of eGFR in drug prescriptions.

## Methods

### Design

Qualitative study using semi-structured interviews.

### Research and study team

The research team was composed of three: LL substitute general practitioner, MJ associate lecturer (Nantes University) and general practitioner, and JPF senior registrar (Nantes University) and general practitioner. Both LL and JPF were novices in conducting a qualitative study. They were therefore assisted by MJ who was experienced in qualitative research. Interviews were run by JPF and LL. The interviewers and participants did not know each other before the start of the study, except for one participant who worked in the same practice as JPF. JPF had integrated eGFR in his prescriptions since 2015; LL and MJ had never integrated eGFR in theirs. The various characteristics of the three interviewers facilitated the analysis, in particular the interpretation of implicit data, since both interviewers and interviewees were physicians (Hardy and Jourdain, 2016).

### Study design

#### Theoretical framework

Data analysis was based on the grounded theory approach, underpinned by a comprehensive perspective of interactionist orientation (analysis of the logic of action and representations of practices) (Morissette, 2011).

#### Selection of participants

Widespread recruitment of physicians working in France and integrating eGFR in their drug prescriptions was initially foreseen. Having interviewed 20 primary care pharmacists of the Nantes area, we noted that the number of physicians integrating eGFR in their prescriptions was marginal. We therefore diversified the sources and recruitment territories: a purposive sample of physicians (regardless of their medical specialty) integrating eGFR in their prescriptions was constituted using messages sent on medical forums, Twitter®, diffusion of electronic mail, direct contact and was completed with snowball sample. All the physicians who responded favourably were included, excepting one who was in favour but who, in the end, did not integrate eGFR in his prescriptions. We aimed at maximum variation on the following criteria: age, sex, type of exercise, being a student supervisor or not.

#### Context

Since recruitment was national, the interviews were conducted using Skype®, telephone calls or face-to-face interviews. Interviews were recorded, transcribed verbatim and checked for accuracy. They were conducted in the consulting rooms or homes of the physicians, as they wished. No non-participants were present.

Before the interview, the researcher asked each participant to provide their first or last five drug prescriptions of their last day of practice, in order to discuss them during the interview.

#### Data collection

A semi-structured interview guide had been elaborated by the research team (Supplemental Table 1). The guide was modified and enriched following the analyses of the first three interviews (modifications are listed in Supplemental Table 1). The guide was composed of six questions. For each of the questions, the points to be discussed were listed. The first question requested that the physician described his/her personal background, and was aimed at encouraging dialogue and collecting elements of profile. The participants were then asked to describe the situations which had led to the prescriptions that they had provided. Using the latter, we discussed the integration or not of eGFR in the prescriptions, the date when the physician started to integrate eGFR in his/her prescriptions, and whether this had changed anything for the patient, pharmacist or the physician him/herself. We then asked the physician for his/her characteristics. And, finally, we asked whether he/she knew other physicians who integrated eGFR in their drug prescriptions.

The interviews took place from February 2016 to April 2017 (duration: 14–44 min, median: 32 min). No interview was repeated and no transcription was returned to the participant. A logbook was kept by LL, in which he collected interview notes and the information from the work sessions.

The sample size was submitted to the principle of data saturation, apprehended by the absence of new ideas emerging from the analysis of new interviews.

Oral informed consent was obtained from each participant before the interview. No ethics committee approval was necessary according to French law (CNGE ethics committee, IRB00010804, advice n°27091852).

#### Analysis

Analyses were conducted in an iterative process. Each verbatim was manually coded by at least two researchers, in an independent process. The interviews were compared and codes were checked against each other after each interview. These codes were then discussed, grouped and associated in themes. Explanatory models of the phenomenon studied were then developed consensually. A final interview was used to check the models for consistency. No software was used. Non-verbal behaviour was not formally collected and analysed. There was no feedback to participants.

## Results

The characteristics of the physicians participating in the study are presented in Table 1. Eight interviews were conducted via Skype®, three by phone and one face-to-face.

**Table 1.** Characteristics of the physicians and interviews

Participant identification	Age (years)	Gender	Type of practice	Place of practice	Years in practice	Student supervisor	Medical software	Estimator integrated	Mode of interview
E1	51	M	Group	Suburban	19	No	Medistory®	MDRD	Skype®
E2	34	F	Group	Urban	5	Yes	Medistory®	CKD-EPI	Skype®
E3	55	M	Group	Urban	20	Yes	Hellodoc®	CG and MDRD	Skype®
E4	31	M	Hospital	Urban	1-2	Yes	Hospital’s software	CG	Telephone
E5	36	F	Group	Urban	2	No	Hellodoc®	CKD-EPI	Telephone
E6	38	F	Group	Suburban	6	Yes	Medistory®	CKD-EPI	Skype®
E7	56	F	Solo	Urban	30	No	Hellodoc®	CG and MDRD	Skype®
E8	36	M	Group	Urban	<1	No	Monlogicielmedical.com®	CKD or MDRD	Skype®
E9	62	M	Group	Suburban	36	Yes	Medistory®	Creatinine, MDRD, and CKD-EPI	Telephone
E10	59	M	Group	Urban	33	Yes	Medistory®	CKD-EPI	Skype®
E11	56	M	Solo	Suburban	27	Yes	Medistory®	MDRD	Skype®
E12	36	M	Group	Urban	5	Yes	Medistory®	CKD-EPI and CG	Face to face

MDRD: Modification of Diet in Renal Disease, CG: Cockcroft–Gault, CKD-EPI: Chronic Kidney Disease Epidemiology Collaboration

Data saturation was considered as reached after 12 interviews.

### Initiation of the process of integrating eGFR in prescriptions

#### Training as key driver

A concrete training course initiated the process of integrating eGFR in medical prescriptions. This course was part of an approach to collectively improve physicians’ habits. A training course was collective for three of the physicians: it was part of a continuing education programme on CKD organised by a general practitioners’ learned society. Another multi-professional training course was organised by biologists with general practitioners and pharmacists. E6: ‘*As a result, we have talked about the pertinence of inscribing the GFR in the prescription, […] and some of [the pharmacists] said: well, that might good for us to see the kidney function’*.

Several physicians had taken a six-month internship in nephrology during their residency. It was even the subject of a medical degree (MD) thesis for one of them. For one of the physicians, the link between his/her internship in nephrology and the subject of the interview had not been made beforehand.

Other initial and continuing education training courses were axed on medical prescriptions for the elderly. Last, a medical software training course provided the opportunity to initiate a discussion on the users’ forum. E11: ‘*training that [E10] had to follow on [medical software], and [medical software] has a forum, and there was a discussion on that. There are formulas that circulate to integrate kidney function’*.

#### Personal and founding experience of an adverse drug reaction

Situations that led to adverse drug reactions were identified as founding in the verbatim of physicians. A physician described a serious adverse event that could have been avoided: E4 ‘*So you have this woman who wasn’t too bad and who, because she was with renal insufficiency, and she is contraindicated for this drug, this hadn’t been monitored correctly, and bang, she had a haemorrhagic shock and died because of it’*.

#### A need for coordination in the framework of patient safety

One of the physicians integrated eGFR following a phone call from a pharmacist. The physician had prescribed an antibiotic at a dose adapted to the stage of the patients’ eGFR, and the pharmacist found the dose too low. The physician had in fact adequately decreased the dose ‘*It’s easier for them to have the reply directly on the prescription’*.

A physician was concerned by the prescriptions of physicians he replaced, emphasising on the drug interaction between a nonsteroidal anti-inflammatory drug (NSAID) and an angiotensin-converting enzyme inhibitor (ACEi) in the elderly. E8: ‘*And perhaps I’m the contrary to certain physicians who I have replaced and for whom kidney function seemed to be the eleventh planet of the solar system’*.

#### A peer community

Belonging to a community gave rise to a synergy in adopting this new habit. All the physicians configured their medical software themselves and some of them found help through forums. A physician stated he had integrated eGFR on impulse from a quality peer group. E9: ‘*That’s when [in the quality peer group] I implemented it, but I had already thought of doing so. That’s often the case, but one needs the trigger’*. Twitter® permitted a physician to participate in a discussion on the integrations of eGFR in prescriptions. Pharmacists following Twitter® appeared highly in favour of this habit, which in turn prompted physicians to implement the habit. E8: ‘*I’d followed conversations on Twitter® during which some pharmacists appeared to appreciate information on kidney function of the patient’*.

### A rich underpinning theory

Physicians reported numerous elements of the theory that underpinned their aims in integrating eGFR in their drug prescriptions, as well as its modalities.

#### Aims

During the interviews, three axes of motivation became apparent.

#### Safety measures

The principal aim of the physicians, stated by all of them, was to allow a control by the dispensing pharmacists. In their views, the physician was not ‘*infallible*’ (E2, E4), and consequently wanted the analysis of the pharmacist (qualified as ‘*safeguard*’) in order to correct any potential errors. E1: *‘I said to myself that if I got it wrong, the pharmacists would call me’*. The notion of cooperation with other healthcare professionals included emergency physicians, radiologists and anaesthetists.

Several physicians, when integrating eGFR, talked of a ‘*visual alert*’ (E9), ‘*automatic alert*’ (E4) or ‘*reminder*’ (E3) when writing out prescriptions.

A physician raised the question of his legal protection regarding safe prescriptions.

#### For the patients

The purpose of this process of integration was to replace the patient in ‘*the centre of his/her health’*. E4: ‘*The idea is to say; well my doctor said “be careful with the kidneys”. I think that it also allows the patient to reintegrate his/her treatment among the health professionals’*. When ageing, the patient could note that his/her kidney function was declining and the number of drugs increasing, and consequently the risk of nephrotoxicity would be accentuated.

#### The prescription as a reflection of professionalism

The prescription was not limited to a list of drugs. Physicians enhanced their prescriptions with various data: weight, BMI and/or dietary advice. Integration of eGFR in the prescription might represent a conspicuous sign of quality of practice. They wished to show their responsibility and attached a particular importance to the image reflected by their prescriptions, E12: ‘*What I feel is important is the image. The prescription, the layout and the heading, that’s the image’*.

#### Modalities

Some physicians integrated eGFR in their prescriptions for all their patients. The other targeted patients included those with a MDRD equation <60 mL/min/1.73 m^2^, and patients aged over 50 years (E10). One physician (E11) also wanted to do so for all patients aged over 50 years, but finally only among the elderly polymedicated patients, those aged over 70 years or with CKD (E7). Nevertheless, the criteria of integration in these targeted patients were not clarified (E7).

Integration of eGFR was computerised for most of the physicians; the latter having configured their medical software. Some manually integrated the eGFR. The choice of the eGFR equation varied among physicians, the CKD-EPI equation was preferred. Two physicians modified the configuration of their medical software so that the date the eGFR was calculated was integrated to the drug prescription, alongside the type of equation chosen.

The physicians spontaneously underlined the importance of this approach for the following drugs: oral antidiabetics (metformin), NSAIDs, direct oral anticoagulants, ACEIs or antibiotics (without further precision). Paracetamol was mentioned by the physicians several times.

### A disappointing practice

The physicians’ initiative was full of hope. However, the feedback appeared to fall short of their expectations.

#### A change for the physicians: a motivation to improve their habits regarding safe prescribing

The physicians claimed that the integration of eGFR in their prescriptions had not notably changed their usual practice. Some physicians reported paying more attention to the dose of drugs administered and have become more reluctant to prescribe certain drugs, such as NSAIDs. Physicians were comforted, knowing that the pharmacist would also check the prescription. Certain physicians claimed that this habit had allowed them to correct a prescription or change a drug (E8): ‘… *correct a prescription that was not called into question and say “she has a clearance of 20, perhaps we should space out the administration of one drug or another”*’.

Finally, some other physicians reported not having changed their habits, since the verification was made before drawing up the prescription: ‘*I generally try to detect it before prescribing* (E10)’. Some even claimed that they did not need the integration (E3): *‘It’s true that up until recently I didn’t feel the need to, so that means I can do my job without inscribing it’*.

#### Feedback from pharmacists

None of the physicians reported having had any contact from a primary care pharmacist wishing to correct a drug prescription because of a non-adapted eGFR. Certain primary care pharmacists would have reported that that was not part of their competences. E3: ‘*the exchanges that we had (…) were more to the order of: “no, anyway I’m just a pharmacist, I dispense drugs, I’m surely not going to question a physician, he knows what he’s doing”’*. Other primary care pharmacists’ reactions reported varied from ‘great perplexity’ (E10) to obvious disinterest. ‘*And the two pharmacists said “… if you want to include it, if that’s what you like, that’s fine, but in any event, we won’t even look at it”’* (E3). The physicians were unfazed by this absence of feedback. E1: ‘*to date they haven’t called me. Perhaps that’ll happen; one mustn’t lose hope’*. E12: ‘*I think that if they’re not interested; then they should become interested’*.

#### Patients’ reactions

The patients would not remark the presence of eGFR on their prescriptions. E7: ‘*they don’t even know that I included it’*. It could have led to reactions in some, such as annoyance or anxiety. Integration of eGFR in prescriptions would sometimes enable patient’s education. E11: ‘*It helps to explain what it refers to, to explain that when one has a kidney that’s not working, one isn’t always aware of it. And then drive the point home regarding NSAIDs that the patients purchase here and there’*.

#### Barriers

Various barriers to integrating eGFR in prescriptions may have limited the extension of this habit. The barrier was often linked to the medical software. Some medical software appeared more adapted than others to the integration in the prescriptions, but required the user’s competence. ‘*The problem is that my software is ill-conceived, at least as far as that is concerned’*.

Some spoke of their concern in not overcharging the prescription, E8: ‘*but I’m not sure that overloading the prescription with this type of equation is really necessary’* or the additional work required, E4 (concerning colleagues): ‘*they can also see the attention that it may have regarding adverse drug reactions […], even though some of them say: “oh it’s long, it means extra work”’*.

The lack of time to update the medical files including eGFR was also underlined by some, E8: ‘*it was a clearance that dated, so it’s true that yesterday, during the consultation, I could have updated the file, looked at the last clearance and noted it, but I didn’t’*.

#### Failure

Despite integration of the eGFR in the prescription, a patient with kidney failure was injected a contrast product.

### Habit typology

A typology of habits was drawn up.

#### The leader

From the start, he had always wanted to practice general practice. He integrated eGFR in his prescriptions, despite the reticence of primary care pharmacists. He inspired other physicians interviewed in this type of initiative; E11: ‘*And then I’d say it was the “harmful” contact with E10 (laughter), we note things in the prescriptions, because he’s full of good ideas’*. He’s involved in the teaching of not only students but also physicians.

#### The ‘geek’

To modify the configuration of a medical software, one has to master it, and some physicians present themselves as computer ‘experts’ (E9): *‘I’ve medical websites, I’ve 3 home pages, 3 Twitter accounts and I created the website […]’*. We often noted computer jargon during the interviews, such as ‘macro’, ‘biometry’, ‘configuring’ or ‘formulae (for configuring)’.

#### The nephrology expert

The geriatrician is more often confronted with CKD than the general practitioner. Prevention of adverse drug reactions is a major concern in daily practice, and also research and teaching, E4: ‘*I try to develop the habit when I’m giving a course on adverse drug reactions. I tell them that in fact in this type of prescriptions they should integrate it’*.

#### The mixed expert

The mixed expert is trained in nephrology (six-month internship) and has written his MD thesis on nephrology. He also fully masters the computer, which he compares to a ‘*Ferrari in spare parts, without the manual’*. (E1)

#### The independents

The independents were neither expert in computers, nor in kidney diseases, and above all wanted to inform the radiologist or the pharmacist of the eGFR of their patients. The independents decided to integrate eGFR, independently of their associates, E2: ‘*…they don’t even draw up their prescriptions on the computer. Obviously they don’t inscribe either the age, weight or anything else’*.

#### The followers

The followers generally belong to a community with inherent group dynamics. Nevertheless, they also master computers, without claiming to be experts. A physician, who had followed a discussion on the topic on Twitter®, was sufficiently intrigued to modify the configuration of his medical software. Other physicians integrated eGFR following recommendations promoted in training courses on the elderly or kidney diseases. We noted the terms ‘*innovative’* or ‘*avant-garde’* (E11) among some of them, as if by integrating the eGFR on the prescriptions signified they were in advance of the other physicians, E11: ‘*I’ve followed everything with the [general practitioners’ learned society]. […] There is the quality of the speakers. It’s detailed, very pertinent… And often it is a little too avant-garde with regard to what is proposed’*.

Contrary to the leaders, some physicians integrated the eGFR so as to enhance their professional image as opposed to other professionals, with little feedback, but extremely rewarding for the physician, E12: ‘*A cardiologist told us that our correspondence was super. I became hysterical, I was so pleased’*.

## Discussion

Residency and training, professional experience (including experiences of adverse drug reactions), and the membership in various communities of professionals appear as facilitators for the integration of eGFR in prescriptions. The theoretical aim is above all safe prescribing in order to reduce adverse drug reactions, with the control by a dispensing pharmacist and other healthcare professionals. However, in fact, none of the physicians received any feedback from the pharmacists or other healthcare professionals. In spite of this, they are convinced of the interest of integrating eGFR in prescriptions, and will therefore continue to do so.

### Strengths and weaknesses

The strengths of our study include the selection of a qualitative method with semi-directed interviews (adapted for exploring an emerging and marginal phenomenon), and the diversity of the recruitment methods to reach this target of unconventional physicians.

This study does have some limitations. First, although we planned a purposeful sampling, difficulties in the recruitment of participants led to the necessity of diversifying the recruitment methods that may limit the variation of our sample of participants on selected variables (e.g., place of practice). Relating to the same issue, the recruitment included a physician who was an associate with one of the investigators (JPF). We took into account this particular interactive context. Second, another physician included declared that he integrated eGFR following a discussion on Twitter®. It is possible that this discussion was initiated by our initial recruitment tweet (i.e., possible recruitment contamination). Last, some may question the participation of a non-primary care physician in the study. We did not plan to restrict our sample to the physician’s type of practice, as long as the physician encountered primary care patients, who would later get their prescriptions dispensed by a primary care pharmacist.

### Comparison with existing literature

We noted a great variability in the theoretical modalities of eGFR integration in the prescriptions. This variability is also observed in the literature on primary care in studies apprehending the collaboration of general practitioners, dispensing pharmacists and control of the prescriptions according to the equation, in terms of age (aged over 18 years (Bhardwaja *et al*., 2011), over 65 years (Via-Sosa *et al*., 2013) and over 70 years (Erler *et al*., 2012; Geerts *et al*., 2012), and equations and targets of eGFR (Cockcroft–Gault <50 mL/min (Bhardwaja *et al*., 2011; Erler *et al*., 2012), MDRD <60 mL/min/1.73 m^2^ (Via-Sosa *et al*., 2013), MDRD <40 mL/min/1.73 m^2^ (Joosten *et al*., 2013)). Another element of variability concerned the drugs mentioned. None of the latter mentioned by the participants appear in the Dutch list of 23 drugs for which the physicians must integrate the therapeutic indication and eGFR of the patient in the prescription (The Royal Dutch Medical Association (KNMG), 2013). In the other articles above, we also noted a variability in the targeted drugs considered at risk of adverse drug reactions (diuretics, anti-gout, renin–angiotensin system inhibitors, antibiotics, oral antidiabetics, digoxin or beta-blockers) (Bhardwaja *et al*., 2011; Breton *et al*., 2011; Erler *et al*., 2012; Geerts *et al*., 2012; The Royal Dutch Medical Association (KNMG), 2013; Via-Sosa *et al*., 2013). A consensus on the target populations, target drugs, estimators and frequency of eGFR monitoring appears necessary in order to homogenise the joint physician/pharmacist interventions aimed at reducing adverse drug reactions in patients with CKD in primary care.

Despite the different theoretical intentions of the physicians, the outcomes were considered disappointing. According to the physicians, there was no feedback (even negative feedback) from the pharmacists concerning the analysis of a prescription. This lack of implication of the pharmacists is in opposition to the results of recent surveys conducted in other countries, which suggested that the pharmacists would appreciate an extension to their role in primary care (Butterworth *et al*., 2017) (and more particularly among patients with CKD) (Zhu *et al*., 2014). In the literature, the pharmacist’s access to the eGFR, associated with an analysis of the prescriptions in primary care, significantly reduces prescription errors (Bhardwaja *et al*., 2011; Breton *et al*., 2011; Geerts *et al*., 2012; Joosten *et al*., 2013; Via-Sosa *et al*., 2013). Various motivations may explain this difference in alleged attitude of the dispensing pharmacists reported in our study. First, this is still an extremely marginal habit. The pharmacists may not be accustomed to using such information in routine. Second, it requires extra time for the pharmacist to check the prescription. In a recent study, about 9 min are required by the pharmacist to check a prescription for a patient with a eGFR <40 mL/min/1.73 m^2^ and, together with the physician, make a decision (Joosten *et al*., 2013). Nevertheless, in the same study, the time required was not considered as problematic for the pharmacists, since it only concerned an average of one patient per week. It should be noted that when a pharmacist controls prescriptions of patients with CKD, around 30% of the interventions proposed to the general practitioners are accepted (Joosten *et al*., 2013; Via-Sosa *et al*., 2013).

Among the physicians, a real wish to collaborate, not only with the pharmacists but also with the other healthcare professionals, was observed. Integration of the eGFR in the prescriptions appeared to them as a supplementary tool for improving this collaboration. A systematic review underlined the fact that the collaboration between general practitioners and pharmacists globally improved the management of the patients, without focalising on patients with CKD (Michot *et al*., 2013). In France, a recent decree has recognised these new roles for primary care pharmacists, including extended medical reviews (Arrêté du 9 mars 2018 portant approbation de l’avenant 12 à la convention nationale du 4 mai 2012, organisant les rapports entre les pharmaciens titulaires d’officine et l’assurance maladie, 2018).

### Implications for research and/or practice

The physicians had set up a novel process, despite the absence of scientific evidence. Evolution of the process over time is uncertain. There are several theories of diffusion of the innovation, among which figures that of Rogers who identifies five elements in favour of diffusion of innovation, some of which have been observed in our study (Rogers, 2003):

- *Relative advantage*: the participants considered the integration of the eGFR in the prescription as advantageous (control by the pharmacist, enhanced inter-professional cooperation and prevention of adverse drug reactions).
- *Compatibility*: mastering of computers would be necessary to guarantee the compatibility with the pre-existing systems.
- *Complexity*: the participants put forward that the process is easy to understand.
- *Testability*: modifications in the medical software appear easy according to those interviewed. It also appears that these modifications are also easily reversible, although no participant raised this possibility.
- *Observed effects*: for the time being, the results observed are reserved, which may constitute a limitation for the diffusion of this process.

Rogers also classified the users of an innovation into five categories: the innovators (2.5%), the early adopters (13.5%), the early majority (34%), the late majority (34%) and the latecomers (16%). Two typologies identified in our study can be classified as ‘innovators’: the ‘experts’ (nephrology expert, ‘geek’ and mixed expert) and the ‘independents’; whereas the ‘early adopters’ correspond more to the ‘followers’. In our study, the ‘early majority’ has not yet been reached. If a widespread diffusion of the phenomenon takes place, it will be necessary to evaluate its adoption by the physicians and the pharmacists, together with its future adaptations.

## Conclusion

Primary care pharmacists cannot fully fulfil their roles in preventing adverse drug reactions in patients with CKD without having access to the patients’ eGFR. Integrating eGFR on physicians’ drug prescriptions could be a simple way of communicating this information to pharmacists, other healthcare professionals and patients. Training and professional experience – including founding experiences of adverse drug reactions – and the membership in various communities (in which ‘leaders’ and ‘followers’ have been identified) are key drivers for the integration of eGFR in prescriptions. In practice and for the time being, physicians who do integrate eGFR are disappointed with the lack of feedback from other healthcare professionals. In spite of this, they continue to integrate eGFR and remain convinced of its interest. The study results also highlighted the different types of practice among physicians. All criteria have not yet been met to consider a broader dissemination of this potential innovation.

## Supplementary material

For supplementary material accompanying this paper visit https://doi.org/10.1017/S1463423619000847. click here to view supplementary material
