# Supplementary material for: Processes and determinants of integration of eGFR in physicians’ drug prescriptions: a qualitative study of semi-structured interviews
Source: Prim Health Care Res Dev. 2019 Oct 23;20:e143. doi: 10.1017/S1463423619000847 (PMC6842646; doi:10.1017/S1463423619000847)
Supplement: Supplementary file 1 [file S1463423619000847sup001.doc]

**SUPPLEMENTAL MATERIAL**

Supplemental Table S1- Interview guide

| **1. Ice-breaking questions:** |
| --- |
| Tell me about your professional background.  (How can this approach be linked to the professional background, what trigger could be used, specific courses, specific training?)  (Specify the *residency courses** or eventual supplementary university degrees)  What led you to study general practice? |
| **2. Together, can we look at your five prescriptions?** |
| (Does the estimated kidney function appear in the prescriptions?)  Tell me about these prescriptions. |
| **3. If estimated kidney function is absent in some prescriptions, when do you integrate it in the prescriptions?** |
| **4. What does it change/has it changed when integrating the estimated kidney function? What is it /was it used for?** |
| Tell me about situations in which it changed something. (Note for the interviewer: the change for the physician, pharmacist and *patient** should be specified)  When was the last time you integrated it? (*Ask for a concrete example**) |
| **5. Tell me about the time when you started to integrate the estimated kidney function.** |
| **6. Characteristics of the physician** |
| Age, gender, type of practice (independent, group), place of practice (urban, suburban, rural), years in practice, medical student supervisor or not  Please list the pharmacists in the area (in order to interview them later, specify that it is for a supplementary study)  Type of medical software (type of integration: automatic, manual?)  Which equation is used (CKD-EPI, MDRD, CG)? |
| **7. Do you know any other physicians who integrate estimated kidney function in their prescriptions?** |

* text in italics has been added after analysis of the first three interviews. No further change was made.

**Supplemental Table S2: Consolidated criteria for reporting qualitative studies (COREQ)**

| **Item** | **Description** | **Response** |
| --- | --- | --- |
| **Domain 1: Research team and reflexivity** | | |
| *Personal Characteristics* | | |
| 1. Interviewer/facilitator | Which author/s conducted the interview or focus group? | LL and JPF conducted the interviews. MJ supervised the interviews. |
| 2. Credentials | What were the researcher’s credentials? E.g. PhD, MD | LL: MD student; MJ: MD; JPFMD, PhD |
| 3. Occupation | What was their occupation at the time of the study? | LL: substitute general practitioner, MJ: GP, associate lecturer; JPF: GP, senior registrar |
| 4. Gender | Was the researcher male or female? | LL: male; MJ: female ,JPF-Male |
| 5. Experience and training | What experience or training did the researcher have? | LL and JPF were novices in conducting a qualitative study. MJ was experienced in qualitative research |
| *Relationship with participants* | | |
| 6. Relationship established | Was a relationship established prior to study commencement? | The physicians were firstly contacted by Twitter, email or phone by LL. He then explained them the study and asked them for their willingness to participate. |
| 7. Participant knowledge of the interviewer | What did the participants know about the researcher? e.g. personal goals, reasons for doing the research | LL and MJ: A relationship with participants was not established prior to study commencement.  JPF practiced in the same general setting as one participant. |
| 8. Interviewer characteristics | What characteristics were reported about the interviewer/facilitator? e.g. Bias, assumptions, reasons and interests in the research topic | JPF had integrated eGFR in his prescriptions since 2015; LL had never integrated eGFR in theirs. |
| **Domain 2: study design** | | |
| *Theoretical framework* | | |
| 9. Methodological orientation and Theory | What methodological orientation was stated to underpin the study? e.g. grounded theory, discourse analysis, ethnography, phenomenology, content analysis | Descriptive qualitative study based on the grounded theory approach, underpinned by a comprehensive perspective of interactionist orientation (analysis of the logic of action and representations of practices). |
| *Participant selection* | | |
| 10. Sampling | How were participants selected? e.g. purposive, convenience, consecutive, snowball | Convenience sampling was used. |
| 11. Method of approach | How were participants approached? e.g. face-to-face, telephone, mail, email | Messages sent on medical forums, Twitter®, diffusion of electronic mail, direct contact and snowball sample. |
| 12. Sample size | How many participants were in the study? | 11 participants were included in the study. |
| 13. Non-participation | How many people refused to participate or dropped out? Reasons? | One participant was contacted who was in favour but who, in the end, did not integrate eGFR in his prescriptions. |
| *Setting* | | |
| 14. Setting of data collection | Where was the data collected? e.g. home, clinic, workplace | interviews were conducted using Skype®, telephone calls or face-to-face interviews.  They were conducted in the consulting rooms or homes of the physicians interviewed, as they wished. |
| 15. Presence of non-participants | Was anyone else present besides the participants and researchers? | No non-participants were present. |
| 16. Description of sample | What are the important characteristics of the sample? e.g. demographic data | Age, gender, type of practice, place of practice, years in practice, student supervisor, eGFR estimator are reported. |
| *Data collection* | | |
| 17. Interview guide | Were questions, prompts, guides provided by the authors? Was it pilot tested? | A semi-structured interview guide had been elaborated by the research team. The guide was modified and enriched following the analyses of the first interviews. The interview guide is available in Supplemental table 1. |
| 18. Repeat interviews | Were repeat interviews carried out? If yes, how many? | Repeat interviews were not carried out. |
| 19. Audio/visual recording | Did the research use audio or visual recording to collect the data? | Audio-recording was used. |
| 20. Field notes | Were field notes made during and/or after the interview or focus group? | A logbook was kept by LL. |
| 21. Duration | What was the duration of the interviews or focus group? | 14 to 44 minutes, median: 32 minutes |
| 22. Data saturation | Was data saturation discussed? | Yes, data saturation was discussed. See Methods. |
| 23. Transcripts returned | Were transcripts returned to participants for comment and/or correction? | No. The transcripts were not returned to participants. |
| **Domain 3: analysis and findings** | | |
| *Data analysis* | | |
| 24. Number of data coders | How many data coders coded the data? | LL, JPF and MJ coded the data. |
| 25. Description of the coding tree | Did authors provide a description of the coding tree? | A description of the coding tree is available from the authors. |
| 26. Derivation of themes | Were themes identified in advance or derived from the data? | Themes were identified from the data. |
| 27. Software | What software, if applicable, was used to manage the data? | Manual coding was used. |
| 28. Participant checking | Did participants provide feedback on the findings? | The participants did not provide feedback on the findings. |
| *Reporting* | | |
| 29. Quotations presented | Were participant quotations presented to illustrate the themes / findings? Was each quotation identified? e.g. participant number | Yes. Each quotation is identified by participant number. |
| 30. Data and findings consistent | Was there consistency between the data presented and the findings? | We believe that there is consistency between the data and findings. |
| 31. Clarity of major themes | Were major themes clearly presented in the findings? | The major themes are clearly presented. |
| 32. Clarity of minor themes | Is there a description of diverse cases or discussion of minor themes? | Minor themes were not included. |
